# The Interplay of Servant Leader and Interpersonal Trust in Predicting Employee-Based Brand Equity: Moderating Role of Ethical Work Climate

**DOI:** 10.3389/fpsyg.2022.905862

**Published:** 2022-06-14

**Authors:** Shaoting Zhang, Shaohua Guo

**Affiliations:** ^1^School of Marxism, Zhengzhou University of Light Industry, Zhengzhou, China; ^2^School of Social Service and Development, Zhengzhou Normal University, Zhengzhou, China

**Keywords:** employee brand based equity, servant leadership, interpersonal trust, ethical work climate, work culture

## Abstract

Although servant leadership may be equipped to provide a leadership model that addresses the issues of the modern workforce, little literature is available regarding the relationship between servant leadership and employee brand-based equity. This study contends to address this gap for which data have been collected from the service industry under a cross-sectional research design by distributing 410 questionnaires among the participants, out of which 337 were received back. After discarding the partially filled and incomplete responses, the useable responses were 314. Data were analyzed via the Smart PLS approach by applying the structural equation modeling technique. Results indicate that servant leadership directly increased the employee-based brand equity by the mediating role of interpersonal trust. However, this study has not established the moderating role of an ethical work climate.

## Introduction

Employee-based brand equity (EBBE) is a fairly new concept that was first used in 1999. It has been used as the subject of numerous books and articles (Keller, 1999; Thomson et al., 1999). Due to the growing importance of internal branding, this concept is expanding to different kinds of corporate brands, including services and business-to-business (B2B) and non-profit industries. The importance of media and marketing is lower in these markets, while the importance of human interaction is greater (Chen et al., 2014; Schwarz et al., 2016). Employees are an integral part of the human resources of any organization, so their mutual interaction plays a significant role in shaping the performance of an organization. It is also generalized that the employees are the brand messengers, so they contribute to creating strong brand equity of respective organizations (Schmidt and Baumgarth, 2018).

Enough scientific work has looked into the relationship between internal brand management (IBM) and brand performance (BP). The majority of studies indicate that IBM has a positive impact on BP (Baumgarth, 2009; Burmann et al., 2009). This indicates that improved BP is a possible outcome of EBBE. To develop EBBE in an organization, potential areas of interest are identified as leadership, communication, and human resource management (Wentzel et al., 2010). Therefore, it becomes more important to address how leadership can boost internal brand management. While previously, empirical research was carried out to assess the impact of leadership on communication, less attention was paid to scientific understanding of the influence of leadership on brand equity (Rehmet and Dinnie, 2013; Schmidt and Baumgarth, 2018; Koch and Gyrd-Jones, 2019).

This synergy relates to the phenomenon of hunter-gatherer requirements studied in evolutionary leadership biology by Vugt and Ronay (2013). Vugt and Ronay (2013) reveal that humans are evolving and the leadership in organizations is also evolving, but several hunter-gatherer requirements are not met. Employees follow their leaders intimately in hunter-gatherer communities, so there remains no distinction between leaders’ personal and social selves (Vugt and Ronay, 2013). Till now, businesses are dealing with smaller and hunter-gatherer communities. The massive administrative enterprises require something like a flexible workforce from across the Globe. To achieve this, enterprises require a sense of tribal membership, which modern organizations frequently fail to provide (Vugt and Ronay, 2013). To overcome this deficit, servant leadership provides a culture of social identity among the members. This is achieved by forming teams that resemble brotherhood in hunter-gatherer societies. In this manner, team members support and grow the ability of others (Yoshida et al., 2014).

Servant leadership has the scope to provide a leadership model which addresses the issues of the modern workforce while somehow satisfying our hunter-gatherer desires for affiliation (Eva et al., 2019). Servant leadership is a comprehensive leadership approach that involves followers on numerous levels (e.g., interpersonal, moral, psychological, and intellectual). Hence, servant leadership can provide grounds for the employees to function to their full potential. The primary goal is to build an employees’ workforce based on the leaders’ humanitarian and moral motivations (Jones, 2018). Whenever the wellbeing or progress of employees is prioritized, leaders become more productive and engaged at work. It is also supported by the fact that servant leaders are the ones who consider themselves to be custodians of the organizations they work for van Dierendonck (2010).

Servant leaders strive to increase the financial and other resources given to them. As a result, despite focusing on their employees’ personal development, they do not neglect performance demands. The performance-oriented leadership frequently sacrifices individuals on the altar of revenue and development. In contrast to this kind of leadership, servant leadership focuses on long-term performance (Sendjaya, 2015). Servant leadership research can be divided into two stages. First, the study concentrates on the theoretical evolution of servant leadership, with (Spears’s, 1996; Thakore’s, 2013) works as examples. Second, the investigations focus on building servant leadership metrics and using cross-sectional research to investigate links between servant leadership and consequences.

Researchers are currently working in the model development phase of servant leadership. In this phase, more sophisticated research strategies are being used to go over simple findings to investigate the precursors, mediation pathways, and model parameters of servant leadership (Eva et al., 2019). Authors believe that a study on the impacts of servant leadership is necessary for certain reasons. First, studies have confirmed the empirical and theoretical distinction of servant leadership from the other kinds of leadership based on Graham’s (1991) work which laid the foundation for the creation of servant leadership theory (Schaubroeck et al., 2011; Hoch et al., 2016). Secondly, despite the growing interest in academic research on servant leadership, there is a lack of consistency and coherence in the field of servant leadership and its impacts on the outcomes of employees.

Extensive research in servant leadership can contribute significantly to other fields, such as healthcare, hospitality, and education. In addition, research indicates that servant leadership works well in the non-profit, governmental, and youth domains (Schaubroeck et al., 2011; Waterman, 2011; Eva and Sendjaya, 2013; Neubert et al., 2016). No prior research has ever evaluated the influence of servant leadership on employee-based brand equity. Therefore, the current study fills this gap by assessing the influence of servant leadership on EBBE. There are some factors that help in developing EBBE, which are utilized in the current research as well. Including these factors, interpersonal trust is highlighted. The positive anticipation that everyone will add to his/her general wellbeing without inflicting harm is referred to as interpersonal trust (Latif et al., 2022).

It is assessed that humans’ behavioral patterns are influenced by interpersonal trust (Karahanna et al., 2005; Simpson, 2007). Interpersonal trust is defined by individuals understanding their susceptibility, behavioral risks, and positive expectations from others. This is common in circumstances wherein interpersonal trust plays an important role. People’s fear of being used by others is reduced by interpersonal trust, which encourages collaboration (Yamagishi and Sato, 1986; Mayer et al., 1995; Rousseau, 1998). Seemingly, employees are more inclined to follow social rules if they believe everyone else will follow them. Furthermore, interpersonal trust aids in the formation of community collaborations to respond to tough conditions, as well as aids in the acceptability of future solutions (Afsar et al., 2021; Hasche et al., 2021).

The relationship of employees with their employers is influenced by personal and organizational factors. So, interpersonal trust is an important component of successful and sustainable human relationships in an organization. In organizational management, it is described as an employee’s conviction in the authenticity of another employee (Asamani, 2015). Kim and Park (2019) utilized interpersonal trust as a mediator in the empirical investigation between organizational learning and transformational leadership. The gap arose in evaluating interpersonal trust as a mediator in the context of servant leadership. Therefore, the authors tried to figure out the connecting link between servant leadership and EBBE. Employees in service firms frequently fall into unethical activities, such as taking the company’s equipment, concealing errors throughout the service delivery system, and handling customers inappropriately. This approach can lead to the undermining of the existing product or service quality of their firms.

This example emphasizes the need for creating an ethical work environment to reduce and avoid the wrong doing of employees (Kim and Koo, 2017). Ethical work climate is defined as having predominant impressions of organizational behaviors and processes which include ethical concerns (Victor and Cullen, 1988). Empirical research shows that having an ethical work environment encourages employees to act ethically (Teng et al., 2020). Based on this assumption, the authors tried to find out the moderating impact of the ethical work climate between servant leadership and EBBE. This research addresses certain aspects such as the role of servant leadership in developing employee-based brand equity with the help of interpersonal trust among employees. This research also focuses on the moderating role of ethical work climate in organizations to develop employee-based brand equity.

## Theoretical Support and Hypothesis

### Servant Leadership Theory

Greenleaf’s (1979) work established the concept of servant leadership theory. According to Greenleaf (1979), the fundamental responsibility of the servant leader is to serve. It originates with the fundamental sensation that one who desires to serve should start serving first. Then, by making a conscious decision, one might aim to lead (Farling et al., 1999). Furthermore, Greenleaf argued that leaders who want to lead first, rather than serve, are doing so out of a desire for control, authority, and personal benefit (Rachmawati and Lantu, 2014). It has been suggested that a leader’s attributes, rather than their professional development plan, determine whether they choose to lead or assist first (Russell and Gregory Stone, 2002).

Leadership style demonstrates the focus of leaders on the organization and is inadequate to describe conduct, that is, humanistic or follower-oriented. Hence, servant leadership theory, which is follower-oriented and focused, explains such behavior (Farling et al., 1999). These characteristics define the servant leader, who is influenced by virtues from within (constructs). These ethical constructions shape the mindsets, qualities, and behaviors of servant leaders (van Dierendonck and Nuijten, 2011).

### Servant Leadership Is Positively Related to Employee Brand-Based Equity

Servant leadership is a management strategy for managing people that includes the employees on different levels (e.g., social, moral, psychological, and intellectual). It can enhance the full capabilities of the employees (Lumpkin and Achen, 2018). Most of the other previous researchers also investigated such connections between servant leadership and management integrity in employees (Toor and Ofori, 2009). Servant leadership used to have a direct influence on the development of employee-based brand equity (Hanaysha and Al-Shaikh, 2021).

Based on this hypothesis, this study attempted to investigate the significance of servant leadership, which is a form of leadership oriented toward a positive impact on employee-based brand equity. The study of performance-based investigations was proposed to analyze the impact of servant leadership in establishing employee-based brand equity (Johnson et al., 2018). The growing significance of the brand’s internal foundation might well be linked to the brand concept’s expansion to cover traditionally restricted areas, such as services, companies, and non-profit organizations (Brydges and Pugh, 2021).

The contingent incentive part of transactional and transformational leadership is servant leadership with mission leadership (Brown et al., 2020). Employees are, indeed, key brand components, primary drivers of brand equity, and positive brand communicators (Castañeda-García et al., 2019). Therefore, servant leadership is positively related to employee-based brand equity.

**H1**: *Servant leadership is positively related to employee brand-based equity.*

### Servant Leadership Is Positively Related to Interpersonal Trust

Servant leadership (SEL) is defined as “an idea and practice of leadership that prioritizes the good among those led over through the self-interest of the leader, stressing leader behaviors that focus upon follower growth, and de-emphasizing glorify of the leader (Tuan, 2022).” Servant leaders are those who lead with a concentration on the supporters, with the believers as the primary concern and organizational matters as a secondary concern (Irving, 2011). The servant-leader constructions are attributes, which are described as a person’s excellent moral qualities, general decency, or moral perfection (Gu et al., 2022). According to the studies related to human relations, interpersonal trust is a key component of successful and long-lasting human partnerships (Afridi et al., 2020).

The interpersonal trust may impact human behavioral patterns (Li and Hsu, 2018). Cooperation among people is widespread in situations where interpersonal trust is vital, as described by individuals recognizing their sensitivity, behavioral risks, and favorable expectations of others (Fischer et al., 2019). Interpersonal trust reduces people’s fear of being one of those others, which increases collaboration (Lei et al., 2019). Users appear to be more likely to observe social rules if they feel everyone else should (Castelli and Sarvary, 2021). Moreover, interpersonal trust contributes to the establishment of community partnerships to respond to difficult circumstances, and also facilitates the acceptance and effectiveness of future solutions (Tsai and Hung, 2019).

**H2**: *Servant leadership is positively related to interpersonal trust.*

### Interpersonal Trust Is Positively Related to Employee Brand-Based Equity

According to the social exchange theory, a person may be pleased to develop an exchange connection with others by willingly providing advantages to others first and then anticipating future rewards (Zhang and Liu, 2021). In theory, trust is a necessary component of a social exchange relationship (Boateng et al., 2019). The greater the social exchange connection between the sender and the recipient, the greater the degree of trust regarded by both parties (Greenberg and Dillman, 2021). When an employee chooses to share organizational information, interpersonal trust becomes critical (Hasche et al., 2021).

However, connections between workers and management are essential in the workplace, therefore trust of the supervisor (TOS) should be included when describing employees’ knowledge-sharing habits (Venters and Wood, 2007). Despite the hypothesized influence of supervisor trust, the actual results are equivocal due to potential methodological differences (Tan et al., 2020). It is vital to reconsider the effects of confidence in colleagues as well as trust in supervisors on knowledge-sharing behavior (Usmanova et al., 2021).

**H3**: *Interpersonal trust is positively related to employee brand-based equity.*

### Mediating Relationship Between Servant Leadership and Employee Brand-Based Equity

The influence of the servant style of leadership on job satisfaction and turnover intention is investigated by looking at the consecutive mediating effects of employer brand perception (Bharadwaj et al., 2021). The main goal of this study is to investigate and evaluate how servant leadership impacts an individual’s withdrawal cognitions, that is, turnover intentions, by maintaining a positive organizational image as just an employer brand image in the mind of current employees and therefore by creating an environment in which employees support the government they work for Mostafa et al. (2021). Literature reinforces the theory that an employee’s view of a Servant leadership style leads to a variety of critical organizational outcomes, including organizational commitment (Faraz et al., 2021). Initial empirical data on the function of organizational identity establish its role in moderating the influence of servant leadership on critical organizational outcomes (Lv et al., 2022).

The leadership’s responsibility is to establish a favorable business identity (Lythreatis et al., 2021). Leadership styles have a critical role in transforming a corporate identity into a favorable organizational image (Verma and Kumar, 2021). In general, interpersonal trust is defined as a person’s belief in the sincerity of another individual’s interpersonal trust as something of an empirical mediator (Kistyanto et al., 2021). Even though there was a deficit in analyzing interpersonal trust as a mediator in the framework of servant leadership, we attempted to identify the connecting relationship between servant leadership and employee-based brand equity (Johnson, 2021).

Ethical work climate is defined by Tehranineshat et al. (2020) as a collective employee concept of ethical activities, ethical practices, and ethical procedures that are influenced by two components: the ethical eligibility requirements used for management decision making and the linkage disequilibrium of analysis used as a referent in the ethical judgment procedure (Kim and Vandenberghe, 2020). This article demonstrates the need for establishing an ethical workplace culture to decrease and avoid misbehavior (McKendall et al., 2002).

An ethical work environment is described as “having dominating perceptions of typical organizational behaviors and activities that have ethical substance” (Fein et al., 2021). According to scientific investigations, having an ethical work atmosphere helps people to act ethically (Ariail et al., 2021). We attempted to determine the moderating influence of an ethical work atmosphere on servant leadership and EBBE based on this premise (Khan and Khan, 2021). This research just aims at the function of servant leadership in generating employee-based brand equity with the support of both interpersonal trust and mutual understanding (Jung et al., 2021). As a result, the authors suggest the following research hypotheses:

**H4**: *Interpersonal trust mediates the relationship between servant leadership and employee brand-based equity.*
**H5**: *Ethical work climate moderates the relationship between servant leadership and employee brand-based equity.*

**FIGURE 1:**
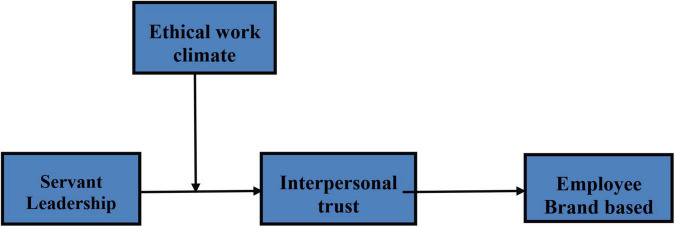
Conceptual framework.

The following conceptual model (Figure 1) has been formed based on the above findings and hypotheses.

## Participants and Methods

We have opted to use a cross-sectional design in this study to obtain data from the participants. The use of cross-sectional research design is common, and in the past, many researchers have used this design in their studies (e.g., see Hao et al., 2020a,b; Nawaz et al., 2021). Further, the survey method is based on the cross-sectional research design, and the data are collected by the convenience sampling technique. This sampling method provides easy access to data collection and is cost-effective in collecting data from a large pool of respondents. Previously, several researchers have used this technique in their studies (Avotra et al., 2021; Yingfei et al., 2021). This technique is based on the non-probability sampling technique, which is most commonly used in cross-sectional studies.

Considering the study’s theoretical orientation, service sector employees were approached for data collection, and the banking industry was selected as the target industry. All bank employees, that is, frontline employees and branch and operation managers, were requested to fill out the survey questionnaires. Before administrating questionnaires to the employees, formal approval was obtained from the in-charge/managers. In addition to this, written informed consent was also obtained from the participants. To boost the motivation of the survey participants, we offered them a discounted meal coupon. Approaching a reasonable audience for data collection is crucial in survey-based research; thus, we have followed a well-known and well-established benchmark of sample size recommended by Tehranineshat et al. (2020) and set minimum sample size of 384. Several researchers have used this criterion to devise a reasonable sample size (Bashir et al., 2020; Wu et al., 2022).

To be safer, we distributed 410 questionnaires among the participants and asked them to fill the questionnaire at their ease. A follow-up message was sent to them after 1 week, and after that, they were approached again for the collection of questionnaires. Of the distributed questionnaires, 337 were received back. After discarding the partially filled and incomplete responses, the useable responses were 314. In cross-sectional studies, the issue of common method biases could arise, and it can shatter the findings. This study attempted to reduce the common method bias by following various measures. Previous researchers have used different methods to address this issue, from using negative questions/reverse-coded questions to changing the place of scale items in questionnaires. So, we used negative questions to restrict the participants from responding in a monotonic manner. To increase the participants’ confidence, we ensured them that this study was for educational purposes and is used only for research purposes. In addition to this, we changed the place of scale items so that respondents could not develop a correlation among the study constructs.

### Scales/Measurement

The Likert scale provides a meaningful and helpful way to record perception. Usually, a five-point Likert Scale is used in the cross-sectional studies, and this study followed the five-point Likert Scale with a score ranging from 5 to 1, where 5 indicates strongly agree and 1 denotes strongly disagree. The predictor in this study is operationalized based on 13 items developed previously (Dennis and Bocarnea, 2005). A sample item for this scale includes, “The level of trust my leader places in me increases my commitment to the organization,” and “My leader trusts me to keep a secret.” This scale illustrates the leader’s behavior in terms of their focus as a steward of the organization, increasing subordinate interactions as a mission of responsibility. Previously, this scale has been used by other researchers (Latif et al., 2022).

The mediating variable of this study (interpersonal trust) is operationalized based on a six-item scale developed previously (Cook and Wall, 1980). This scale is divided into two dimensions: one indicates trust in peers, while the other dimension indicates trust in management. Both dimensions are measured based on three items each. A sample item for trust in peers includes, “I can trust the people I work with to lend me a hand if I need it,” and sample items for trust in management include, “Management at my firm is sincere in its attempts to meet the employees’ point of view.” The outcome variable in this study is measured using a five-item scale developed previously (Baumgarth and Schmidt, 2010). This scale measures the employee’s perception regarding their brand-based equity. The sample item for this scale is “I am aware that everything I say or do can affect the brand image.” The scale used to measure the moderating variable, that is, the ethical climate, was obtained from a previous study (Arnaud, 2010). This scale originally contained 18 items with 6 dimensions. We selected the most relevant dimensions of this scale, that is, moral awareness, collective moral motivation, and character. Three items for each dimension and nine items have been used for this scale in this study.

## Results and Hypothesis Testing

### Assessment of Measurement and Structural Model

Owning to the complex nature of the framework, we utilized a multivariate data analysis tool. We analyzed the data using the structural equation modeling (SEM) technique and the Smart PLS 3.9 software (Yingfei et al., 2021). Smart PLS, in this regard, was the best available choice because it deals with the non-normal data very comfortably, and the issue of small sample size is dealt with very conveniently through bootstrapping approach (Hair et al., 2017). As in the case of brand-based equity in terms of employees, the theory is under development, and it was best suited to use the partial least square approach (Hair et al., 2014).

Initially, the measurement model was assessed for reliability and validity (see Table 1). Reliability was assessed through “Cronbach’s alpha, rho-a, and composite reliability (CR),” while validity was assessed through convergent and discriminant validity. First, the reliability parameters were found within the acceptable range (i.e., >0.60), which indicates that reliability was established. The reliability values indicated that higher reliability was observed in the case of servant leadership, which was 0.931. Similarly, other reliability parameters, such as rho-A and composite reliability, indicated a good level of reliability (Hair et al., 2011; Bashir et al., 2020).

**TABLE 1:** Reliability and convergent validity of the study constructs.

Construct	Indicator	FL	VIF	Cronbach’s alpha	rho_A	Composite reliability	AVE
EBBE	BE1	0.730	1.221	0.814	0.853	0.862	0.555
	BE2	0.704	2.077				
	BE3	0.766	2.952				
	BE4	0.714	1.422				
	BE5	0.807	3.979				
IPT	IPT1	0.895	2.898	0.898	0.981	0.921	0.703
	IPT3	0.932	4.576				
	IPT4	0.770	2.071				
	IPT5	0.826	5.201				
	IPT6	0.753	4.099				
SL	SL1	0.770	2.102	0.931	0.941	0.942	0.619
	SL10	0.783	3.579				
	SL11	0.764	3.778				
	SL12	0.740	3.183				
	SL2	0.881	4.666				
	SL4	0.792	3.546				
	SL6	0.870	3.564				
	SL7	0.680	1.784				
	SL8	0.814	4.790				
	SL9	0.756	3.423				

*EBBE, Employee brand-based equity; IPT, Interpersonal trust; SL, Servant leadership.*

**FIGURE 2:**
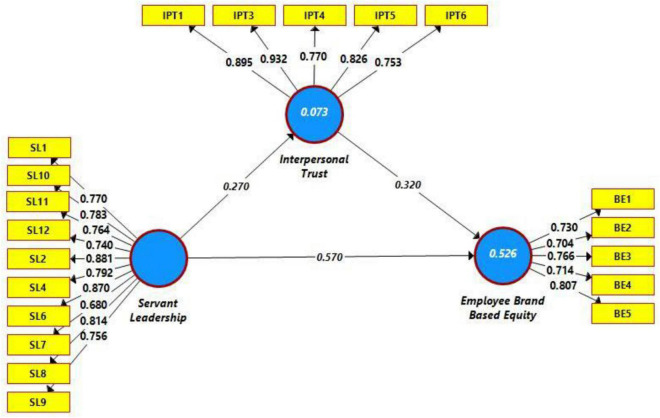
Path estimates.

Indicator reliability was assessed based on outer loadings. Table 1 illustrates the indicator reliability. The obtained values were within the acceptable limits (>0.708), except for BE-2 and SL-7 values, which were slightly low compared to the benchmark value. Indicators with these lower loadings were retained because the AVE of the respective construct was higher than the desired value (>0.50). AVE of employee-based brand equity was 0.555, while AVE of servant leadership was 0.619. Thus, these constructs indicated more than 50% variance. However, some items for servant leadership and interpersonal trust were dropped due to poor outer loadings (values less than 0.40). One item from the interpersonal trust (IPT-2) and three items (SL-3, SL-5, and SL-13) were dropped from the analysis due to poor outer loadings (Figure 2). Thus, both outer loadings and AVE of the constructs (illustrated in Table 1) were within the acceptable range, and convergent validity was established (Mela and Kopalle, 2002).

While discussing discriminant validity, we used two well-established criteria, that is, Fornell and Larcker (1981) and HTMT ratios (Hair et al., 2017). First, Table 2 illustrates that the square root of the AVE of all the three constructs is higher than the correlational values in the respective column and row (diagonal). These findings established discriminant validity through the Fornell and Larcker (1981) criteria (Sarstedt et al., 2014).

**TABLE 2:** Discriminant validity [Fornell and Larcker (1981) criteria].

Construct	Employee brand based equity	Interpersonal trust	Servant leadership
Employee brand based equity	0.745	–	–
Interpersonal trust	0.474	0.838	–
Servant leadership	0.656	0.270	0.787

With regard to HTMT ratios, Table 3 illustrates that HTMT ratios in all columns are less than 0.90, thus establishing the discriminant validity. In this case, both the conservative and liberal criteria of HTMT are observed because the values of HTMT are also less than 0.85 (Table 4).

**TABLE 3:** Discriminant validity (HTMT).

Construct	Employee brand based equity	Interpersonal trust	Servant leadership
Employee brand based equity	–	–	–
Interpersonal trust	0.524	–	–
Servant leadership	0.615	0.260	–

**TABLE 4:** Direct, indirect, and total path estimates.

Direct path	Beta	SD	*t*	*p*
Interpersonal trust → employee brand based equity	0.320	0.038	8.325	0.000
Servant leadership → employee brand based equity	0.570	0.034	16.616	0.000
Servant leadership → interpersonal trust	0.270	0.054	4.999	0.000
**Specified indirect path**				
Servant leadership → interpersonal trust → employee brand based equity	0.086	0.019	4.461	0.000
**Total path**				
Servant leadership → employee brand based equity	0.656	0.029	22.956	0.000

**FIGURE 3:**
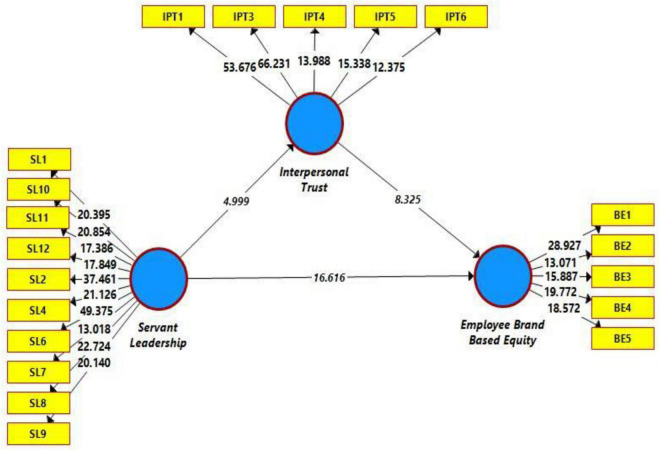
Path significance.

Additionally, we also assessed the issue of multicollinearity (assessed through VIF), and all the observed values were within the acceptable range (Table 1). Additionally, we also evaluated the coefficient of determination (R^2^) and effect size (f^2^) as a tool to assess the structural model before testing the hypothesis. First, the effect size was good (0.079–0.635). These effect sizes are large enough and indicate a reasonable effect size to assess the model’s fitness. Moreover, we also evaluated the percentage of change by calculating R^2^. In this regard, 53% change was observed in employee-based brand equity through servant leadership and interpersonal trust, while 7% change was observed in interpersonal trust due to servant leadership (Hair et al., 2006; Bashir et al., 2020), which is evident from Figures 2, 3 (Hair et al., 2017). The predictive relevance (Q^2^) was found to be satisfactory because the obtained value of Q^2^ was larger than zero (Geisser, 1975).

Hypothesis testing was done in two ways: the direct and mediating hypotheses were tested through SEM, while the moderation hypothesis was tested based on Process Macro. Table 5 illustrates the results of hypotheses testing. The first hypothesis of this study, which is related to the relationship between servant leadership and employee-based brand equity, was found to be statistically significant because the *t* and *p* values were found to be significant (*t* = 16.616, *p* < 0.00, and *B* = 0.570), also illustrated in Figure 3. The value of coefficient of regression indicates that one unit change in servant leadership will cause a change of 0.570 units in employee-based brand equity, thus H1 is supported through the statistical approach. Similarly, the impact of servant leadership on interpersonal trust is statistically significant (*t* = 4.999, *p* < 0.00, and *B* = 0.270). Here, the coefficient value indicates that one unit change in servant leadership will cause a change of 0.270 units in interpersonal trust, thus H2 is supported. Similarly, the third hypothesis of this study was related to the impact of interpersonal trust on employee-based brand equity, which was found to be statistically significant (as evident from p and t statistics). The values of p and t were within the threshold limits, thus H3 is also supported. When considering mediation indirect effect, that is, servant leadership–interpersonal trust–employee brand-based equity, the indirect impact was statistically significant, indicating that servant leadership promoted interpersonal trust, which further increased employee brand-based equity.

**TABLE 5:** Hypotheses testing.

Hypotheses		Coefficient (Beta)	SD	*t*	*p*	Status
H1	Servant leadership → employee brand based equity	0.570	0.034	16.616	0.000	Supported
H2	Servant leadership → interpersonal trust	0.270	0.054	4.999	0.000	Supported
H3	Interpersonal trust → employee brand based equity	0.320	0.038	8.325	0.000	Supported

**Mediation hypothesis**		**Coefficient (Beta)**	**SD**	***t***	***p***	**Status**

H4	Servant leadership → interpersonal trust → employee brand based equity	0.086	0.019	4.461	0.000	Supported

**Moderation hypothesis**		**Coefficient (Beta)**	***f***	***Df1***	***Df2***	**Status**

H5	Servant leadership × ethical climate → employee brand based equity	0.006	2.499	1.000	347.000	Not supported

## Discussion

Several researchers discovered empirical and conceptual evidence for the diverse effectiveness of servant leadership. In recent years, this body of empirical research has grown significantly, resulting in non-transparency and complexity in the domain of servant leadership. A rise in performance is one potential effect of servant leadership that has been widely explored (Liden et al., 2013; Huang et al., 2016). The evaluation of performance-based investigations suggested evaluating the role of servant leadership in building employee-based brand equity. Growing evidence from recent literature suggested that internal brand management, which develops employee-based brand equity, is the contributor to outer brand performance (Baumgarth, 2009; Burmann et al., 2009). It was also noted that leadership along with human resources is a major player in managing a brand internally, and it could develop the brand more firmly (Wentzel et al., 2010).

Based on the above-mentioned findings, this study tried to explore the role of servant leadership, which is a type of leadership toward the development of employee-based brand equity. This research also focused to assess the mediating impact of interpersonal trust between servant leadership and employee-based brand equity. Furthermore, the moderating role of ethical work climate was also assessed in the current research. The results provided significant foundations for the suggested concept. For this reason, direct impacts, such as servant leadership to employee-based brand equity, servant leadership to interpersonal trust, and interpersonal trust to employee-based equity, were analyzed. Similarly, indirect effects were also a focus of the study, which included the mediating role of interpersonal trust between servant leadership and employee-based brand equity and the moderating role of ethical work climate for developing employee-based brand equity.

The direct results indicated that servant leadership directly influenced the development of employee-based brand equity. These results are possibly the reason that leadership at this level motivates the employees to work hard for their organizations. Servant leadership can understand and address the concerns of modern employees of current times and satisfies our hunter-gatherer desires for affiliation (Eva et al., 2019). The effects of servant leadership on interpersonal trust and the role of interpersonal trust on employee-based brand equity were also significant in the current study. These results indicated that servant leadership not only influences the overall performance of their organizations, but can also influence the employees in such a way that a deficit of trust is controlled by them among employees which leads to better performance, and this performance translates into the development of employee-based brand equity.

Servant leadership is a schematic management approach to encourage the employees involved in various domains (e.g., interpersonal, moral, psychological, and intellectual) and promote the full potential of employees (Jones, 2018). Some of the previous scholars also evaluated such relationships between servant leadership and interpersonal trust in employees and got similar kinds of results (Politis and Politis, 2017). The indirect effects of interpersonal trust as a mediator between servant leadership and employee-based brand equity showed a significant association, indicating that trust among employees is a helping tool in such relationships. Once the employees develop trust among themselves, a sense of rivalry is eliminated from their minds, and this condition helps the business to grow.

A few scholars of the past also evaluated the mediating role of interpersonal trust in different situations and got similar results (Kim and Park, 2019), which support our model. The moderating role of ethical work climate was also tested in this study, which proved to not regulate these relationships for employee-based brand equity development. This could be due to the reason that servant leadership had a strong influence on employees, which leads to EBBE with a special focus on interpersonal trust among employees. Therefore, the ethical work climate did not regulate these relationships, although some scholars suggested that it could moderate the relationships from various perspectives (Teng et al., 2020).

## Conclusion

Empirical findings of this study indicate that servant leadership promotes employee-based brand equity. Thus, servant leadership, a management strategy, can help manage individuals in the workplace at various levels (e.g., social, moral, psychological, and intellectual). Thus, servant leadership directly influences the development of employee-based brand equity and positively impacts EBBE. Moreover, it can also be drawn from the empirical evidence of this study that servant leadership increases interpersonal trust. The reason might be that servant leaders try to promote an individual’s attributes, such as excellent moral qualities, general decency, and moral perfection. Thus, developing such characteristics is essential to promote mutual trust, and employees tend to create interpersonal trust. In human relations research, interpersonal trust is critical for successful and long-lasting human partnerships.

Similarly, based on this study, interpersonal trust has the potency to increase employee brand-based equity. Because trust is an essential element of a social exchange relationship (Boateng et al., 2019), the higher the social exchange connection between the two parties, the greater the degree of trust regarded by both parties (Greenberg and Dillman, 2021). Thus, interpersonal trust promotes employee-based brand equity. However, the moderating role of ethical work climate has not been proved in this study in promoting interpersonal trust through servant leadership. Thus, it can be concluded that organizations should try to promote a culture of servant leadership to create an environment that would nurture interpersonal trust and employee brand-based equity.

## Theoretical and Practical Implications

From a theoretical perspective, this study touched on the line of employee-based brand equity through servant leadership and interpersonal trust. First, this study tends to add to the existing body of knowledge by investigating the role of servant leadership in promoting employee brand-based equity. This is the first study that has documented the role of servant leadership in developing and shaping employee-based brand equity. Although overwhelming literature regarding servant leadership theory is available in healthcare, hospitality, and education sectors, no prior research has investigated the influence of servant leadership on employee-based brand equity. Second, this study added to the literature about interpersonal trust by quantifying the impact of servant leadership on interpersonal trust. Third, this study investigated the role of ethical work climate in predicting interpersonal trust based on servant leadership. Thus, this study extended the literature on servant leadership by establishing the empirical and theoretical distinction of servant leadership theory.

Moreover, this study increases the coherence in the servant leadership area. Therefore, the current research would fill this gap by assessing the influence of servant leadership on EBBE. The positive anticipation that everyone will add to one’s general wellbeing without inflicting harm is referred to as interpersonal trust. From a practical point of view, this study posits that organizations should focus on promoting servant leadership to increase employee brand-based equity. Moreover, it will also increase interpersonal trust because interpersonal trust is an essential component of successful and sustainable human relationships in the workplace. Moreover, promoting interpersonal trust in the workplace can foster positive behaviors and increase organizational learning. The development of trust in the workplace can shape more positive behaviors, such as engagement, commitment, and job satisfaction.

## Limitations of the Study

This study has some limitations too. For instance, the first limitation of this study is its cross-sectional nature. Thus, conducting longitudinal research on these variables can provide important insights in the future. Second, increasing the sample size in the future would also be an important area of research. Third, this study used only one mediator, and other potential mediators can also be used to investigate the impact of servant leadership in the future, in addition the helping behaviors in the shape of organizational citizenship behavior (individual and organizational) can also be a mediating variable. Moreover, the role of ethical work climate has not been proved in this study, and hence considering other moderating variables would provide deeper insights in the future. In this regard, Islamic work ethics can also be considered as a moderating variable. Similarly, organizational culture can also be a potential moderator in this regard.

Other leadership styles, such as transformational/transactional and paternalistic leadership styles, can also be a predictor variable in future studies. Similarly, employee-based brand equity can be tested in the shape of its dimensions in future studies covering the dimension of brand allegiance, brand consistent behavior, and brand endorsement. Investigating the impact of servant leadership on brand allegiance, brand endorsement, and brand-consistent behavior would be an interesting area for future research. This study considered only banking sector employees while including other service sectors such as hospitality and retail service sectors would provide important insights. Similarly, adding other sectors into future studies might provide deeper insights. In future studies, researchers can compare employee-based brand equity in different organizations.

## Data Availability Statement

The original contributions presented in this study are included in the article/supplementary material, further inquiries can be directed to the corresponding author.

## Ethics Statement

The studies involving human participants were reviewed and approved by the Zhengzhou University of Light Industry, China. The patients/participants provided their written informed consent to participate in this study. The study was conducted according to the guidelines of the Declaration of Helsinki.

## Author Contributions

SZ conceived and designed the concept and wrote the manuscript. SG collected the data. Both authors read and agreed to the published version of the manuscript.

## Conflict of Interest

The authors declare that the research was conducted in the absence of any commercial or financial relationships that could be construed as a potential conflict of interest.

## Publisher’s Note

All claims expressed in this article are solely those of the authors and do not necessarily represent those of their affiliated organizations, or those of the publisher, the editors and the reviewers. Any product that may be evaluated in this article, or claim that may be made by its manufacturer, is not guaranteed or endorsed by the publisher.
